# Functional significance of cholesterol metabolism in cancer: from threat to treatment

**DOI:** 10.1038/s12276-023-01079-w

**Published:** 2023-09-01

**Authors:** Mingming Xiao, Jin Xu, Wei Wang, Bo Zhang, Jiang Liu, Jialin Li, Hang Xu, Yingjun Zhao, Xianjun Yu, Si Shi

**Affiliations:** 1 Department of Pancreatic Surgery, Fudan University Shanghai Cancer Center, Shanghai, 200032 China; 2 Department of Oncology, Shanghai Medical College, Fudan University, Shanghai, 200032 China; 3 Shanghai Pancreatic Center Institute, Shanghai, 200032 China; 4 Pancreatic Center Institute, Fudan University, Shanghai, 200032 China; 5 Institutes of Biomedical Sciences, Shanghai Medical College, Fudan University, Shanghai, 200032 China

**Keywords:** Cancer, Cancer

## Abstract

Cholesterol is an essential structural component of membranes that contributes to membrane integrity and fluidity. Cholesterol homeostasis plays a critical role in the maintenance of cellular activities. Recently, increasing evidence has indicated that cholesterol is a major determinant by modulating cell signaling events governing the hallmarks of cancer. Numerous studies have shown the functional significance of cholesterol metabolism in tumorigenesis, cancer progression and metastasis through its regulatory effects on the immune response, ferroptosis, autophagy, cell stemness, and the DNA damage response. Here, we summarize recent literature describing cholesterol metabolism in cancer cells, including the cholesterol metabolism pathways and the mutual regulatory mechanisms involved in cancer progression and cholesterol metabolism. We also discuss various drugs targeting cholesterol metabolism to suggest new strategies for cancer treatment.

## Cancer: Changes in cholesterol metabolism

Emerging evidence suggests that changes in cholesterol metabolism can be involved in the onset and progression of cancer, opening avenues towards better understanding of cancer and new treatment options. Cholesterol is an essential structural component of cell membranes, important for maintaining optimal fluidity of the membrane under varying conditions. Si Shi, Xianjun Yu and colleagues at Fudan University Shanghai Cancer Center, China, review recent research into cholesterol metabolism in cancers, including cellular regulatory pathways involving cholesterol that are also implicated in cancer progression. The influence of cholesterol metabolism on cancer has been linked to effects on several key physiological processes, including the immune response, regulated cell death, recycling of cellular components, DNA repair, and the activities of stem cells. The authors consider the potential of drugs known to influence cholesterol metabolism in anti-cancer therapy.

## Introduction

Cholesterol is an important lipid in mammals and an essential component of the membrane. In addition to its role as a membrane constituent, cholesterol is a precursor of bile acids and steroid hormones. Cholesterol and its derivatives are critical for cellular functions. Dyshomeostasis of cholesterol is one of the hallmarks of cancer^1^. In cancer cells, cholesterol uptake and synthesis rates are usually increased, leading to abnormal metabolism^2,3^. The efflux and esterification of cholesterol affect the formation of tumor cells^4,5^. Oxysterols, derivatives of cholesterol, are also involved in tumorigenesis^6^. Recent studies have shown that the regulation of cholesterol level with the respect to the tumor microenvironment (TME) plays an important role in tumor cell proliferation^7^. In addition, ferroptosis, autophagy, tumor cell, and immune cell stemness, and the cellular DNA damage response (DDR) are regulated through cholesterol metabolism^8–10^. Cholesterol metabolism plays an important role in tumorigenesis and progression, and therefore, targeting cholesterol metabolism has become a new direction in the treatment of cancer.

In this review, we summarize recent literature regarding cholesterol metabolism in cancer cells, including discussion on the cholesterol metabolism process and metabolites, the functional significance of cholesterol metabolism in cancer cells and mutual regulatory mechanisms. We also discuss various drugs targeting cholesterol metabolism to establish new strategies for cancer treatment.

## Overview of cholesterol metabolism

**Fig. 1:**
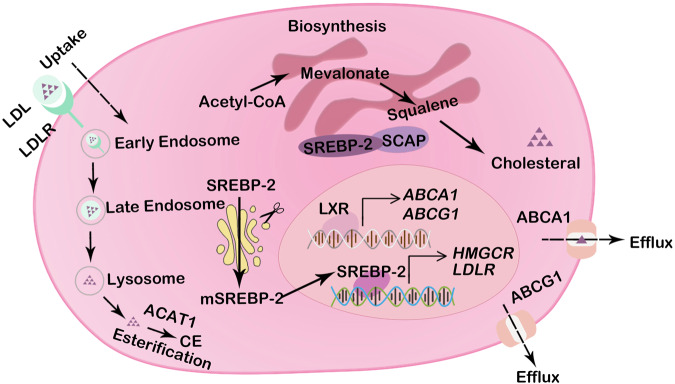
The landscape of cholesterol homeostasis regulation. Major pathways of cholesterol metabolism in cancer cells include cholesterol biosynthesis, uptake, efflux, and esterification. Cholesterol is synthesized through the mevalonate pathway in the ER in an extremely complex process: It starts with acetyl-CoA, then mevalonate and squalene are synthesized, and finally cholesterol is generated. Cholesterol uptake depends on the LDLR-mediated endocytosis pathway. Cholesterol efflux is mediated by ABCA1 and ABCG1. Cholesterol is a precursor of CEs, which are generated via the action ACAT1. The transcription of cholesterol metabolism-related genes (such as HMGCR, LDLR, ABCA1, and ABCG1) is mediated by mature SREBP-2. SREBP-2 is cleaved to form mature SREBP-2 in the Golgi apparatus.

The process of cholesterol metabolism involves an acetyl coenzyme A (acetyl-CoA)-based synthesis pathway, cholesterol uptake, efflux, and esterification (Fig. 1).

### De novo cholesterol biosynthesis

**Fig. 2:**
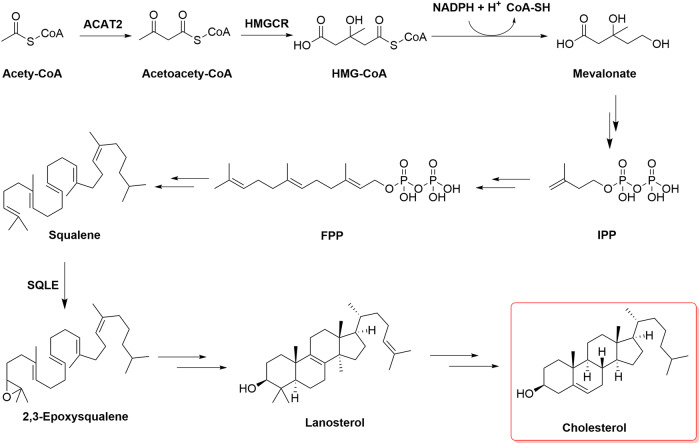
De novo biosynthesis of cholesterol. Cholesterol is synthesized through a series of ~30 reactions. The substrate in the mevalonate pathway is acetyl-CoA, which is condensed into acetoacetyl-CoA. Acetoacetyl-CoA engages with another molecule of acetyl-CoA in a reaction catalyzed by HMG-CoA synthase, forming HMG-CoA. HMGCR catalyzes the reduction of HMG-CoA to yield MVA. MVA undergoes a three-step enzymatic reaction involving phosphorylation and decarboxylation to produce isopentenyl pyrophosphate (IPP). A series of enzymatic reactions converts IPP to farnesyl pyrophosphate (FPP), and two molecules of FPP condense into squalene in a reaction catalyzed by squalene synthase. Squalene is oxidized to be 2,3-epoxysqualene by SQLE and then cyclized to lanosterol, which is eventually converted to cholesterol.

Cholesterol is synthesized through the mevalonate pathway via an extremely complex process involving the synthesis of 3-hydroxy-3-methylglutaryl-CoA (HMG-CoA), mevalonic acid (MVA), and squalene and their subsequent conversion into other molecules. These biosynthetic processes, for which acetyl-CoA is the starting material, are energy consuming and depend on ATP and NADPH (Fig. 2). Generally, two molecules of acetyl-CoA are condensed into acetoacetyl-CoA in a process catalyzed by cytosolic thiolase, which binds with one molecule of acetyl-CoA in a reaction catalyzed by HMG-CoA synthase to generate HMG-CoA. HMG-CoA reductase (HMGCR), the primary rate-limiting enzyme, catalyzes the reduction of HMG-CoA to MVA, which requires the consumption of two NADPH molecules. MVA undergoes a three-step enzymatic reaction involving phosphorylation and decarboxylation to yield isopentenyl pyrophosphate (IPP). A series of enzymatic reactions in the cytoplasm converts IPP to farnesyl pyrophosphate (FPP), and two molecules of FPP are condensed into squalene in a reaction catalyzed by squalene synthase. Squalene is oxidized to 2,3-epoxysqualene by squalene epoxidase (SQLE) and then cyclized to lanosterol, which is eventually converted to cholesterol through a complex reaction in the endoplasmic reticulum (ER). Newly synthesized cholesterol in the ER is directly or indirectly transported to the cell membrane through the Golgi apparatus^11^.

**Fig. 3:**
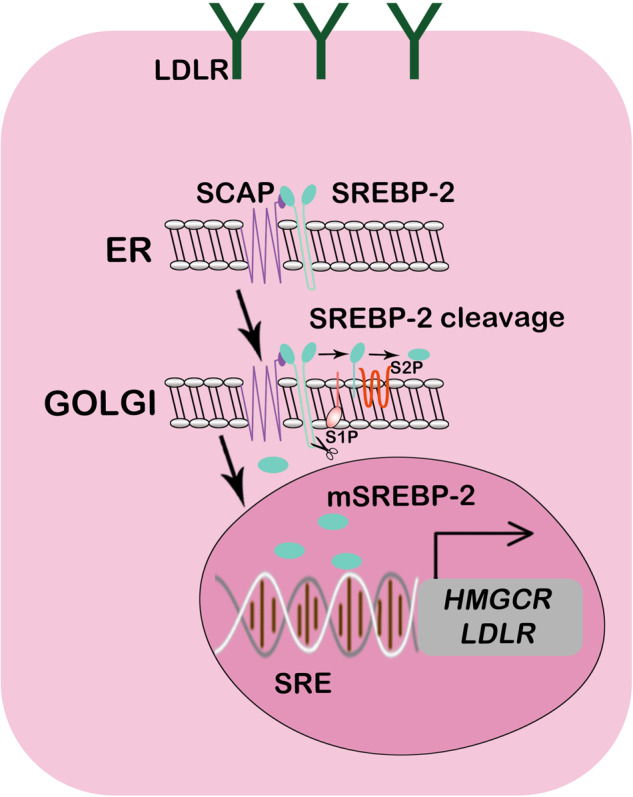
Transcriptional regulation of cholesterol biosynthesis. SCAP interacts with SREBP and mediates the translocation of SREBP from the ER to the Golgi apparatus, where it is cleaved by proteases. Active fragments of SREBP in the nucleus bind to the SRE sequence in HMGCR or another cholesterol synthesis-related enzyme promotor region, thereby initiating gene transcription.

Sterol regulatory element binding proteins (SREBPs) are transcription factors that play central roles in modulating cholesterol biosynthesis^12^. SREBPs constitute a class of membrane proteins in the bHLH-Zip superfamily; they are anchored to the ER and nucleus through their “basic helix-loop-helix-leucine zipper” (bHLH-Zip) motif^13,14^. Three members of the SREBP family have been identified: SREBP-1a, SREBP-1c, and SREBP-2. SREBP-1a and SREBP-1c mainly regulate the expression of enzymes related to fatty acid, triglyceride, and glucose metabolism, while SREBP-2 mainly regulates cholesterol metabolism (Fig. 3). SREBP-2, synthesized in the ER, is a nonfunctional precursor that forms a complex with SREBP cleavage-activating protein (SCAP) to coregulate intracellular cholesterol levels. SREBP2 must be translocated from the ER to the Golgi apparatus, where it is released from the membrane by site 1 protease (S1P) and site 2 protease (S2P)^15^. After sequential processing by S1P and S2P, SREBP-2 loses the N-terminal fragment containing the bHLH-Zip region^16^. The processed SREBP2 enters the nucleus as a homodimer and attaches to the sterol regulatory element (SRE) sequence in the promoters of target genes, such as HMGCR and SQLE (encoding squalene monooxygenase)^17,18^.

### Cholesterol uptake

**Fig. 4:**
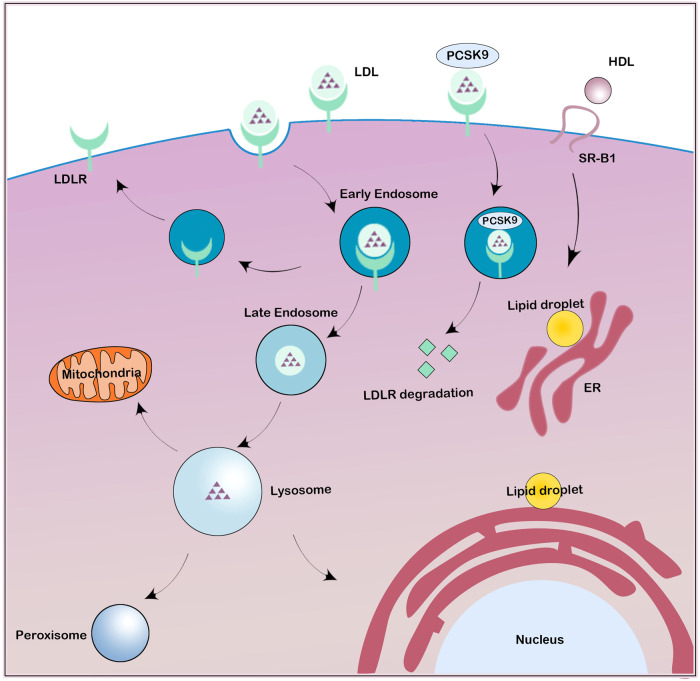
LDLR-mediated LDL endocytosis and SR-B1-mediated HDL selective uptake. LDL particles combine with LDLR in the cell membrane and enter cells through endocytosis. Free cholesterol is then dissociated from the LDLR in the lysosome. Unbound LDLR is recycled to the cell membrane. LDLR is degraded in lysosomes in the presence of PCSK9. HDL particles bind to SR-B1 on the cell membrane. HDL-derived cholesterol and cholesteryl esters can be delivered into the cell via the hydrophobic channels mediated by SR-B1 and further to other organelles, such as the endoplasmic reticulum and mitochondria.

In addition to de novo cholesterol biosynthesis in the ER, cells mainly acquire cholesterol from the receptor-mediated uptake of exogenous cholesterol, including low-density lipoprotein (LDL) acquisition through the endocytosis pathway and high-density lipoprotein (HDL) acquisition through the selective uptake pathway (Fig. 4). These pathways jointly regulate cellular cholesterol levels^19,20^.

LDL receptor (LDLR)-mediated LDL endocytosis is the most important mechanism underlying plasma cholesterol clearance. LDL particles bind to an LDLR in the cell membrane and eventually reaches the lysosome after internalization where it releases free cholesterol through acid lipase hydrolysis. Receptor-mediated HDL cholesteryl ester uptake requires the involvement of HDL receptor scavenger receptor B-type I (SR-BI), which is a member of the class B family of scavenger receptors^20^. SR-BI is a lipophilic channel in the plasma membrane, and through its extracellular domain, SR-BI binds to HDL cholesteryl esters and selectively delivers them into the cell. The extracellular loop of SR-BI contains six highly conserved cysteine residues that are critical to SR-BI activity^21,22^. Notably, mice with SR-BI mutations presented with increased plasma cholesterol levels^23^, and inhibition of SR-BI glycosylation led to defective selective uptake of HDL cholesteryl esters^24^.

### Cholesterol efflux and esterification

**Fig. 5:**
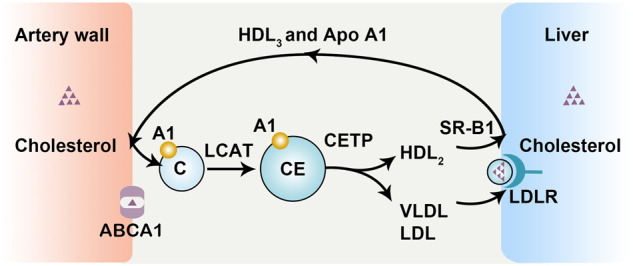
HDL and the reverse cholesterol transport. ABCA1 mediates cellular HDL efflux, and HDL surface cholesterol is esterified by LCAT. With an increase in cholesterol esters, HDL is converted into HDL2 and HDL3. HDL is finally absorbed into hepatocytes in the presence of the HDL receptor SR-B1. In addition, the cholesterol esters in HDL can be transferred to VLDL by CETP and produce LDL.

Dietary cholesterol is absorbed from the gastrointestinal tract, where cholesterol and triglycerides form chylomicrons. In the blood circulatory system, chylomicrons are modified into celiac remnants, which are then transported to the liver. In the liver, very low-density lipoprotein (VLDL) particles of lipids and cholesterol are secreted by hepatocytes, and these particles are further modified into LDL in the blood circulation and then transported to peripheral cells. Excess cholesterol in peripheral cells is released as HDL-C, which triggers the reverse transport of cholesterol back to the liver (Fig. 5).

Cholesterol efflux is the most important step in reverse cholesterol transport. There are four ways in which cholesterol is released from cells: (1) passive diffusion of cholesterol by mature HDL particles, (2) SR-B1-mediated facilitated diffusion, (3) ATP-binding cassette (ABC) transporter subfamily A member 1 (ABCA1)-mediated efflux with ApoA1, and (4) ABC subfamily G (ABCG)-mediated efflux with mature HDL^25^. Free cholesterol (FC) released from the plasma membrane undergoes passive aqueous diffusion mediated via HDL and is driven by the cholesterol gradient, which is maintained through esterification of HDL surface cholesterol through the action of lecithin–cholesterol acyltransferase (LCAT)^25^.

The plasma contains not only FC but also esterified cholesterol. Cholesterol esterification into cholesteryl esters is mediated by acyl-CoA: cholesterol acyltransferase (ACATs). Of the two ACATs ACAT1 and ACAT2, ACAT1 is expressed in most human tissue cells. Cholesterol esters synthesized by ACAT1 are usually stored in lipid droplets. In contrast, ACAT2 is expressed in a development- and species-specific manner. ACAT2 is highly expressed in the human intestine and in the infant liver, and the cholesteryl esters synthesized by ACAT2 are mainly transported by lipoproteins, which can be secreted out of cells^26,27^. Studies have shown that the expression and activity of ACAT-1 are increased in breast, pancreatic, and glioblastoma tumor cells and that they promote cholesteryl ester production^28^. Excessive cholesteryl ester production activates SREBP1, which promotes tumor metastasis. Inhibition of ACAT-1 production leads to inhibited glioblastoma growth and prostate cancer cell invasiveness^5,29^.

### Cholesterol oxygenation and oxysterols

In addition to cholesteryl esters, cholesterol can be converted into oxysterols directly through autooxidation in the presence of reactive oxygen species (ROS) or via enzymatic processes. Oxysterols are found in low or very low concentrations in the human body and mainly exist in oxidized lipoproteins^30^, which modulate the fluidity of the cell membrane and exhibit other cellular functions. Cholesterol oxygenation usually occurs in the steroid backbone or aliphatic sidechain^6^. Oxidation in the side chain generates 27-hydroxycholesterol (27-HC), 25-hydroxycholesterol (25-HC), 24-hydroxycholesterol (24-HC), and 22-hydrocholesterol (22-HC). Backbone oxidation generates 7-ketocholesterol (7-KC), 5,6α/β-epoxycholesterol (5,6α-EC/5,6β-EC), and 7α/β-hydroxycholesterol (7α/β-HC). Oxysterol production on the side chain is mainly mediated through enzymatic processes, which are mainly catalyzed by the cytochrome p450 (CYP) family of enzymes^31,32^. 27-HC is generated by CYP27A1 and catabolized by CYP7B1. CYP46A1 catalyzes cholesterol metabolism to yield 24(S)-HC. 22(R)-HC is generated by CYP11A1. However, 25-HC is generated by cholesterol-25-hydroxylase, not the CYP family. In addition, lipid peroxidation indirectly generates oxysterols, such as B-ring oxysterols^33^.

## The regulation of cholesterol metabolism in cancer

**Fig. 6:**
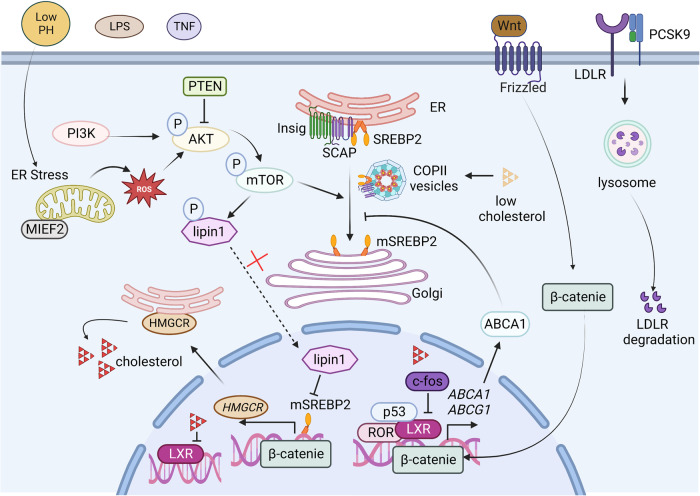
Internal and external factor regulation of cholesterol metabolism. The SCAP-SREBP2 complex is trafficked in COPII vesicles from the ER to the Golgi for proteolytic activation of SREBP2 when the intracellular cholesterol is low. PI3K/AKT, mTOR, and PTEN are critical for SREBP activation. MIEF2 enhances lipid biosynthesis by increasing mitochondrial reactive oxygen species (ROS) production and promoting the subsequent activation of the AKT/mTOR signaling pathway. The Wnt-β-catenin and p53 pathways are critical for the transcription of MVA pathway-related genes. c-Fos inhibits LXR signaling and increases the production of cholesterol. PCSK9 induces the degradation of LDLR. Cholesterol metabolites such as oxysterols function as endogenous ligands for LXRs, suppressing the transcriptional program to inhibit LDL uptake and promote cholesterol efflux, thereby maintaining cholesterol homeostasis. External factors such as low pH, LPS, and TNF can affect cholesterol metabolism.

Internal and external factors regulation of cholesterol metabolism, driving altered cholesterol pathways in cancer cells is critical (Fig. 6). Internal factors such as some signaling pathway molecules or cholesterol itself affect cholesterol metabolism by regulating the activity of SREBP and LXR. External factors such as the acidification and inflammatory of the tumor microenvironment (TME) could also affect cholesterol metabolism.

### Intercellular molecules mediate cholesterol metabolism

SREBP activation plays a central role in the abnormal cholesterol metabolism of tumor cells. SREBP is inhibited by ER cholesterol, Insig protein, and SCAP. Cholesterol concentrations regulate the evacuation of the SREBP2 precursor from the ER through SCAP, which binds SREBP2 via its C-terminal domain. SCAP detects and reacts to changes in ER cholesterol to control the switch between open and closed by modulating its binding to COPII-coated vesicles. The SCAP-SREBP2 complex is sorted into COPII vesicles and transported from the ER to the Golgi, where the proteolytic activation of SREBP2 is triggered when the intracellular cholesterol is low^34^. The dynamic balance in intracellular cholesterol levels regulated by SREBPs requires the joint contributions of SCAP, S1P, S2P, and SRE, and any abnormality, such as a mutation, in any one of these participants can lead to the disruption of cholesterol metabolism^35^. 25-Hydroxycholesterol and other oxysterols are much more effective than cholesterol in binding to Insig proteins and promoting Insig binding to SCAP, which triggers ER retention of the SCAP-SREBP2 complex^36^. Under sterol-deficient conditions, Insig1 degradation leads to the dissociation of the SCAP-Insig complex and activation of the SREBP2 pathway, permitting the transcription of downstream genes^37^.

**Fig. 7:**
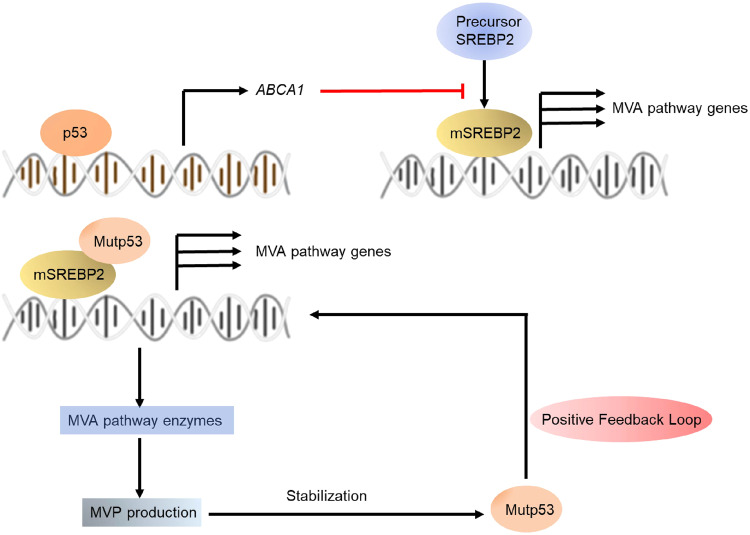
p53-mediated cholesterol metabolism in cancer. p53 increases the expression of the cholesterol efflux transporter ABCA1, repressing SREBP2 maturation and subsequently inhibiting the MVA pathway. Mutp53 promotes mevalonate pathway activation by interacting with SREBPs, increasing SREBP activity. The activated mevalonate pathway increases the levels of MVP, leading to mutp53 stabilization (positive-feedback loop).

In tumor cells, overactivation of the PI3K/AKT signaling and p53-mediated signaling pathways influence SREBP activity. The AKT/mTOR pathway is the most frequently studied de novo cholesterol synthesis pathway in cancer cells. Akt drives SREBP-2 activity and inhibits SREBP-2 degradation, thereby promoting the expression of cholesterol synthesis-related genes^38^. mTOR1 is an important upstream signaling molecule in Akt-induced SREBP activation. mTOR1 promotes nSREBP-2 proteins by phosphorylating and inhibiting the nuclear entrance of lipin 1 and regulating cholesterol trafficking from lysosomes to the ER^39^. Studies have shown that PTEN affects AKT and mTOR activation, thus regulating SREBP-2-mediated target transcription^40^. In addition to the AKT/mTOR pathway, p53 plays an important role in the MVA pathway (Fig. 7). Mutations in p53 promote the mevalonate pathway by interacting with SREBPs and increasing the activity of SREBPs^41^. These mutations induce mevalonate-5-phosphate (MVP) production, and in turn, MVP promotes mutant p53 stabilization, triggering positive feedback regulation of SREBPs^42^. Accumulation and stabilization of mature SREBP2 increase mevalonate pathway enzyme expression in the absence of p53^43^. In addition, p53 increases the expression of the cholesterol efflux transporter ABCA1, thereby repressing SREBP2 maturation and subsequently inhibiting the MVA pathway^41^. Additionally, SREBP2 transcriptional activity is promoted by PIK3CA mutations or growth factors through various mechanisms. In prostate cancer, androgen receptor (AR) signaling mediates SCAP upregulation and promotes SREBP activity^44^. Moreover, the Wnt-β-catenin pathway is activated upon Cilia repression, which promotes the expression of genes involved in the MVA pathway through SREBP-2 interactions^45^. MIEF2 (mitochondrial elongation factor 2) is one of the key regulators of mitochondrial fission. Studies have shown that MIEF2 enhances lipid biosynthesis by increasing mitochondrial reactive oxygen species (ROS) production and subsequent activation of the AKT/mTOR signaling pathway, upregulating the expression of SREBP1 and SREBP2 and their transcriptional targets, such as the lipogenic enzymes ACC1, FASN, SCD1, HMGCS1, and HMGCR. MIEF2 overexpression-mediated mitochondrial dysfunction plays a key role in the reprogramming of lipid metabolism in ovarian cancer cells^46^.

Liver X receptors (LXRs), which form obligate heterodimers with retinoid X receptors (RXRs), are other transcription factors that regulate the expression of ABCA1 and ABCG1^47^. As sensors, LXRs are activated by a high intracellular cholesterol content. The oncogene c-Fos in hepatocytes inhibits LXR signaling and increases the production of cholesterol and cholesterol-derived metabolites, such as oxysterols and bile acids. These effects are associated with increased hepatocellular carcinogenesis^48^. Oxysterols can function as endogenous ligands for LXRs, suppressing a transcriptional program to inhibit LDL uptake and promote cholesterol efflux, thereby maintaining cholesterol homeostasis^49^. Several oxysterols, such as 22(R)-HC, 25-HC, 27-HC, and 24(S)-HC, modulate the activity of LXRs^49,50^. In addition, some oxysterols exert effects by targeting other nuclear receptors, such as RAR-related orphan receptors (RORs), estrogen receptors (ERs), and the glucocorticoid receptor (GR). Different oxysterols show various functions, acting as both agonists or inverse agonists for RORs and thus participate in cholesterol metabolism. 27HC functions as an agonist of RORγ. However, 24(S)-HC and 25-HC have been reported as inverse agonists of RORα and RORγ^51^. Interestingly, a study reported that 25-HC and 22-HC agonistic activity restored RORγ transcriptional activity that had been suppressed by a synthetic inhibitor^52^. In addition, 22(R)-HC, 24(S)-HC, 25-HC, 27-HC, and 7 KC have been reported to regulate ER activities^52^. In addition, 6-oxo-cholestan-3β,5α-diol (OCDO) generated from 5,6-EC has been identified as a GR ligand^53^. OCDO has also reported as a dual GR and LXR ligand. 27-HC is the most abundant oxysterol in the cell membrane and blood. Therefore, it easily crosses the blood‒brain barrier and functions as a “cerebrosterol”. Increasing evidence suggests that 27-HC is important in cancer cells, especially in breast cancer cells^54^. Studies have elucidated that 27-HC activates LXRs to promote the metastasis of ER-positive breast cancer cells^54,55^. However, in endometrial cancer, 27-HC promotes cell proliferation by activating ERs but not LXRs^56^. 25-HC also promotes cell migration and invasion in lung, gastric, and brain cancers by activating a G protein-coupled receptor or the Toll-like receptor 2 (TLR2)/NF-kB pathways and the LXR/interleukin-1B (IL-1B) signaling pathway^56–58^.

In addition to that at the transcriptional level, cholesterol synthesis is also regulated at the translational level. In sterol-rich environments where HMGCR transcription is inhibited, HMGCR synthesis is controlled by mevalonate derivatives. FPP and GGPP are downstream of mevalonate in the cholesterol synthesis pathway, and depletion of mevalonate via HMG-CoA reductase inhibitors reduces the availability of FPP and GGPP^35^, thereby regulating cholesterol synthesis. Cholesterol derivatives, such as 25-hydroxycholesterol or oxygenated cholesterol derivatives such as 27-HC have been shown to reduce the activity of HMGCR^55^.

Cholesterol synthesis-related enzymes, such as HMGCR, are regulated via posttranslational modification, including acetylation, ubiquitination, and phosphorylation. Gp78, TRC8, HRD1, MARCHF6, and RNF145 are E3 ubiquitin ligases that lead to HMGCR degradation^59–62^. HMGCR degradation is induced by sterols, including C4-dimethylated sterols and lanosterol and its C24-saturated derivative 24,25-dihydrolanosterol; however, cholesterol itself does not affect HMGCR itself^63–66^. In addition to ubiquitination, HMGCR is regulated via phosphorylation. In humans, AMPK-mediated HMGCR Ser872 phosphorylation inhibits the activity of HMGCR and decreases cholesterol production^67^.

### Modulation of cholesterol metabolism by the tumor microenvironment (TME)

In addition to its influence on intercellular molecules, the TME is involved in the regulation of cholesterol metabolism. Low pH conditions promote cholesterol biosynthesis^68^. For example, in pancreatic cancer cells at pH 6.8, low-pH-responsive genes mediate SREBP2 nuclear localization, upregulating target gene expression. Under hypoxic conditions and low nutrient concentrations, TME-induced ER stress affects cholesterol metabolism because cholesterol is synthesized in the ER^69^. Moreover, lipopolysaccharide (LPS) and the cytokine TNF enhance cholesterol accumulation by activating SREBP2^70^. HGFs in the liver microenvironment activate cholesterol metabolism through c-Met/PI3K/AKT/mTOR signaling pathways^71^.

### Noncoding RNAs (ncRNAs) and cholesterol homeostasis in cancer

Numerous studies have shown that ncRNAs affect cholesterol homeostasis by influencing cholesterol transport, uptake, and efflux, and these ncRNAs play critical roles in cancer progression. Table 1 shows the ncRNAs affecting gene expression in cholesterol metabolism. MicroRNAs (miRNAs), such as miR-128-1, miR-148a, miR-130b, and miR-301b, which are highly conserved in vertebrates and expressed in many tissues, have been found to play critical regulatory roles in cholesterol homeostasis and transport^72–74^. The target proteins of miRNAs include LDLR, ABCA1 and the insulin receptor (InsR). MiR-185, miR-96, and miR-223 have been reported to regulate the selective uptake of HDL-C by targeting hepatic SR-B1^75^. Specifically, miR-24 targets the SR-B1 3′ untranslated region (UTR) and inhibits SR-B1 expression. miR-24 has been found to promote the expression of genes involved in cholesterol synthesis in an indirect manner^76^. miR-33, encoded by an intron within the SREBF2 gene, is located upstream of ABCA1^77,78^. miR-183 functions as an oncogene targeting the 3′UTR of ABCA1, and reportedly regulates cholesterol efflux in many malignancies^79,80^. miR-26, miR-20a/b, miR-758, and miR-19b have also been reported to regulate cholesterol efflux by targeting ABCA1^81–84^. For example, downregulation of miR-129-5p expression promoted ABCG1 expression and led to chemoresistance in gastric cancer cells^85^. The long noncoding RNA (lncRNA) lncARSR, a regulator of AKT signaling associated with hepatocellular carcinoma (HCC) and renal cell carcinoma (RCC), regulates SREBP-2 expression^86^. Furthermore, in plasma, a high level of the lncRNA HULC, which downregulates miR-9 expression and upregulates RXRA expression during cholesterol synthesis, has been identified as a biomarker of liver cancer^87^. NEAT1 and ANRIL, which are also associated with cholesterol synthesis, are considered biomarkers of non-small cell lung cancer^88,89^. In addition, MALAT1, known to be involved in cholesterol efflux, has been suggested to be a diagnostic indicator of lung cancer^90^.

**Table 1:** Regulation of cholesterol metabolism by noncoding RNA.

ncRNA	Targets	Mechanism	References
miR-128-1	ABCA1, LDLR	Regulation of efflux and uptake	^72^
miR-148a	ABCA1, LDLR	Regulation of efflux and uptake	^72,73^
miR-130b	ABCA1, LDLR	Regulation of efflux and uptake	^72,74^
miR-301b	ABCA1, LDLR	Regulation of efflux and uptake	^72^
miR-185,	SR-B1	Regulation of selective uptake of HDL-c	^74,75^
miR-96	SR-B1	Regulation of selective uptake of HDL-c	^75^
miR-223	SR-B1	Regulation of selective uptake of HDL-c	^75^
miR-24	SR-B1	Regulation of cholesterol uptake	^76^
miR-33	SREBP2, ABCA1	Upstream of ABCA1	^77,78^
miR-183	ABCA1	Regulation cholesterol efflux	^79,80^
miR-26	ABCA1	Regulation cholesterol efflux	^84^
miR-20a/b,	ABCA1	Regulation cholesterol efflux	^81^
miR-758	ABCA1	Regulation cholesterol efflux	^83^
miR-19b	ABCA1	Regulation cholesterol efflux	^82^
miR-129-5p	ABCG1	Upregulation of ABCG1 expression	^85^
lncARSR	SREBP-2, HMGCR	Upregulation of SREBP-2 and HMGCR expression	^86^
HULC	RXRA	Downregulation of miR-9 expression and upregulation of RXRA expression	^87^
NEAT1	miR-342-3p	Modulation of immune cell functions and lipid uptake	^88^
ANRIL	Unknown	Modulation of the inflammatory response	^89^
MALAT1	ABCA1	Regulation of cholesterol accumulation	^90^

## Functional significance of cholesterol metabolism in cancer

**Fig. 8:**
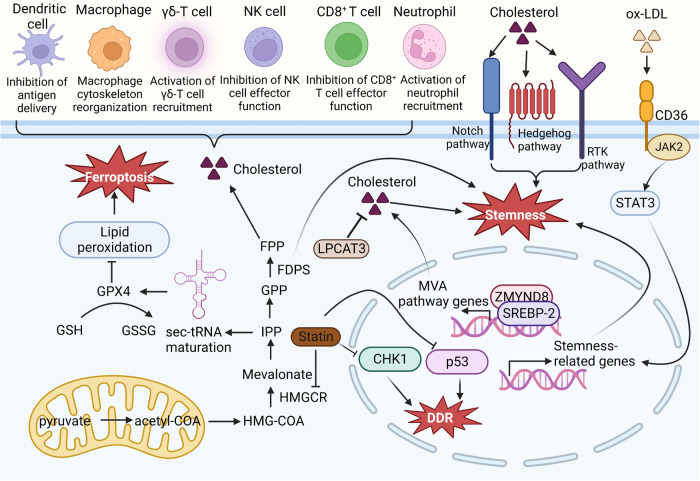
Cholesterol affects tumor cells by modulating immune responses, ferroptosis, tumor cell stemness, and the DNA damage response. Cholesterol is related to various immune cells, including DCs, CD8+ cells, neutrophils, NK cells, macrophages, and γδ-T cells, to regulate the cellular immune response. IPP regulates GPX4 activity and ferroptosis by promoting Sec-tRNA maturation. Cholesterol activates cellular signaling pathways downstream of sonic hedgehog, Notch and receptor tyrosine kinases and promotes stem cell development. In addition, ZMYND8/SREBP2-coordinated enhancer–promoter interactions activate the MVA pathway and promote intestinal stemness. FDPS is critical for cell stemness. Statins, inhibitors of HMGCR, affect the DDR by inhibiting CHK1 and p53 activity.

The reprogramming of lipid metabolism is a hallmark of cancer. Cholesterol, an important component of lipids, is thought to be necessary for cancer cell proliferation and survival. Mechanistically, cholesterol affects tumor cells by modulating immune responses, ferroptosis and autophagy, tumor cell stemness, and the DNA damage response (Fig. 8).

### Cholesterol metabolism regulates immune responses in the cancer context

The interplay between tumor metabolic reprogramming and immune cells is a determining factor in the antitumor immune response. Increasing evidence has suggested that cholesterol metabolism not only plays a key role in tumorigenesis and patient survival but also influences the function of immunity-related molecules^91,92^. High cholesterol levels in tumor cells facilitates tumor cell escape from immune surveillance, and tumor cell-induced increases in the cholesterol level promote the expression of suppressive immune checkpoint genes, thereby inhibiting antitumor effects. Indeed, high cholesterol disrupts the homeostasis of the lipid metabolic network in immune cells, suppressing the immune response. In total, cholesterol metabolism regulates immune responses in three ways: inhibition of antigen presentation, enrichment of immunosuppressive cells (neutrophils and tumor-associated macrophages (TAMs), and regulation of immune-effector cells.

#### Inhibition of antigen presentation and enrichment of immunosuppressive cells

Conditional medium obtained from several cancer cell cultures activated LXR-α signaling on the dendritic cell surface, thereby decreasing CC chemokine receptor-7 (CCR7) expression in these DCs^93^. Cholesterol metabolites are also related to the enrichment of immunosuppressive cells. Cholesterol metabolites, such as 22-HC, 24S-HC, 24-HC, and 27-HC, recruit neutrophils. One study showed that 27-HC promoted breast cancer metastasis by affecting immune cells^94^. Elimination or inhibition of the CYP27A1 enzyme, which is critical to 27-HC biosynthesis, significantly reduced cancer metastasis. The strong effect of 27-HC on metastasis depended on the function of bone marrow immune cells. 27-HC increased the number of polymorphonuclear neutrophils and γδ-T cells at distant metastatic sites and decreased the number of CD8^+^ T cells^94^.

In recent years, considerable clinical and preclinical evidence has shown that TAMs play important roles in tumor growth, invasion, and metastasis. Researchers have conducted in-depth studies on the reprogramming mechanism in TAMs in the TME. They proposed that tumor cell secretion promotes membrane cholesterol efflux from TAMs, which drives TAM reprogramming and promotes tumor growth^95^. Researchers first performed a subgroup analysis to detect the phenotypes of TAMs in a tumor model and found that peripheral blood mononuclear cell-derived (MN-derived) TAMs were gradually replaced by embryonic precursor cell-derived resident macrophages. Moreover, a transcriptome analysis showed that the expression of cholesterol metabolism- and efflux-related genes in TAMs was significantly upregulated during tumor progression. Further experiments revealed that ovarian cancer cells promoted membrane cholesterol efflux by secreting hyaluronic acid (HA), an extracellular matrix component, resulting in the absence of cholesterol-enriched lipid rafts in the cell membrane. These cholesterol-related alterations promoted the onset of IL-4-mediated reprogramming in macrophages. Thus, macrophages acquired an IL-4-induced phenotype, and IFN-γ-related signaling was disrupted, which resulted in the suppression of IFN-γ-induced expression of related genes. Further functional experiments demonstrated that knocking out ABCs prevented TAM reprogramming and inhibited tumor growth.

#### Regulation of immune effector cells

The regulation of cholesterol metabolism-related enzymes has a profound impact on immune effector cells. A typical example in tumors involves the immunomodulatory function of PCSK9, which is related to cholesterol endocytosis into tumor cells. Knocking down or blocking PCSK9 activity through treatment with a specific antibody enhanced the effect of tumor immunotherapy in mice. PCSK9, a serine protease produced predominantly in the liver, induces the degradation of LDLR^96^. Overexpression of PCSK9, which binds to LDLR, resulted in a much lower ability of a patient to eliminate LDL-cholesterol (LDL-C)^97^. In this case, the effects of PCSK9 inhibition in conjunction with an anti-PD-1 antibody treatment lead to a synergistic therapeutic outcome^91^. Knocking out PCSK9 significantly inhibited tumor growth in immunocompetent mice, but this outcome was not observed in immunodeficient mice. Recently, another team found that PCSK9 inhibited LDLR expression in CD8^+^ T cells, thereby repressing the antitumor activity of these CD8^+^ T cells. These data strongly suggest that PCSK9 inhibition may significantly enhance the immune response and suppress tumor growth^98^. Cholesterol metabolism and transporter enzymes exert important effects on the production and activity of immune cells. For example, ACAT1 has been shown to be a regulatory target in the T-cell metabolic pathway^99^. The activation and production of CD8^+^ T cells are required for maintaining the membrane cholesterol content, which is partially regulated by ACAT1. Notably, knocking out ACAT1 in CD8^+^ T cells increased the level of membrane cholesterol, increased T-cell receptor aggregation, promoted T-cell proliferation and activation, and ultimately enhanced their antitumor effects^99^. These studies suggested that cholesterol at high levels exerts immunosuppressive effects. However, the question remains: how does high cholesterol promote the expression of immune checkpoint genes? To answer this question, researchers treated T cells with different concentrations of cholesterol and then used gene chips to analyze the effect. They found that high cholesterol disrupted the lipid metabolic network, which induced ER stress^100^. ER stress led to upregulated expression of XBP1, an ER stress receptor^101^. XBP1 activation, in turn, promoted the expression of immune checkpoint genes, causing T-cell suppression^100^. Recent studies have revealed that XBP1 is a transcription factor involved in cholesterol biosynthesis. ER stress induces XBP1, driving cholesterol biosynthesis by directly binding to the promoters of key genes^102^.

In addition to metabolism-related enzymes, some studies have found that 25-HC inhibits trogocytosis and promotes the antitumor activities of cytotoxic T lymphocytes^103^. In addition, NK cell functions are regulated by cholesterol^104,105^. SREBP is a key factor in regulating energy production in NK cells, which affects NK cell activation^105^.

### Abnormal cholesterol metabolism induces ferroptosis and autophagy in cancer cells

Depletion of glutathione, decreased glutathione peroxidase (GPX4) activity, or reduced cellular antioxidant capacity results in lipid peroxidation and metabolic dysfunction and increased levels of lipid reactive oxygen species (ROS), which trigger ferroptosis^106^. The susceptibility of cells to ferroptosis is closely related to many biological processes, including amino acid, ferritin, and polyunsaturated fatty acid metabolism.

GPX4 is a selenium-containing protein with a selenocysteine active center, and it is required for the function of a specific selenocysteine transporter, selenocysteine transfer RNA (Sec-tRNA)^107^. The addition of IPP to specific adenine sites in Sec-tRNA precursors is necessary for Sec-tRNA maturation; hence, IPP exerts an important effect on ferroptosis. FPP, dolichol, squalene, and coenzyme Q (CoQ), which are IPP derivatives, are also involved in the Sec-tRNA maturation process and exert multiple effects on ferroptosis^108^. The mechanism underlying p53 regulation of ferroptosis may involve its recently recognized role as a key mediator of the MVA pathway. Under metabolic stress, p53 mediates the expression of ABCA1, which is critical for the reverse translocation of cholesterol from the plasma membrane to the ER. This leads to the inactivation of SREBP2 and gene transcription and ultimately inhibits the production of several metabolites, such as squalene and CoQ.

Recently, researchers have found that high cholesterol results in resistance to ferroptosis and increased tumorigenicity and metastasis in breast cancer^8^. Migrating cancer cells can engulf cholesterol in response to stress. Generally, most cancer cells die when exposed to stress, but surviving cancer cells contain high concentrations of cholesterol to counteract the onset of ferroptosis. Cells chronically exposed to 27-HC were shown to cell exhibiting increased cellular uptake and/or biosynthesis of cholesterol^8^. These cells exhibit significantly increased tumorigenic and metastatic capacity, and GPX4 prevents ferroptosis and promotes their metastasis.

Statins, which block the rate-limiting enzymes in the ferroptosis pathway, interfere with the efficient translation of GPX4, increasing cell susceptibility to ferroptosis. Squalene is thought to exert an antiferroptotic effect on cancer cells^109^. Cholesterol is also involved in ferroptosis, and exogenous cholesterol hydroperoxide has been shown to induce cell death in a dose-dependent manner^110^. GPX4 inhibition accelerates ferroptosis induced by cholesterol hydroperoxide, while GPX4 overexpression greatly enhances cell resistance to cholesterol hydroperoxide-induced ferroptosis.

Recent research has found that decreases in cell membrane cholesterol facilitates autophagy initiation protein recruitment and promotes autophagosome synthesis. The cholesterol transporter protein GRAMD1C may inhibit autophagosome synthesis and decrease mitochondrial bioenergetics by regulating cholesterol transport between the cell membrane and endoplasmic reticulum or between the endoplasmic reticulum and mitochondria. Although the study explained the function of the cholesterol transporter protein in autophagy, the roles of cholesterol regulation in autophagosome development and cancer progression remain unknown^111^.

### Cholesterol metabolism and cell stemness in cancer

Cholesterol biosynthesis plays an important role in maintaining tumor stem cells^112^. It can activate cellular signaling pathways downstream of sonic hedgehog, Notch and receptor tyrosine kinases and promote stem cell development. To capitalize on their own robust self-renewal properties, intestinal stem cells require an adequate supply of lipids and cholesterol. Excess dietary cholesterol or enhanced intracellular cholesterol synthesis accelerated tumorigenesis in mice. Zinc finger MYND type 8-containing (ZMYND8), an epigenetic reader, interacts with SREBP2 to activate the MVA pathway and promotes intestinal stemness and tumorigenesis^113,114^. A research group has provided insight into this relationship from the perspective of the regulation of lipid and cholesterol dynamic homeostasis^9^. Inhibition of the phospholipid remodeling enzyme LPCAT3 increased cholesterol saturation in the membrane and stimulated cholesterol biosynthesis, which in turn promoted small intestine stem cell proliferation. Pharmacological blockade of cholesterol synthesis inhibited the development of intestinal crypts in LPCAT3-deficient mice. In contrast, increasing intracellular cholesterol levels stimulated the development of intestinal crypts. Feeding a diet with excess cholesterol or increasing endogenous cholesterol synthesis through SREBP-2 expression promoted small intestine stem cell proliferation in vivo. In addition, the group found that disrupting the LPCAT3-mediated dynamic balance between phospholipid and cholesterol levels significantly increased the tumorigenesis rate in Apc^-/-^ mice.

Recent research has revealed a mechanism through which hypercholesterolemia-induced production of oxidized low-density lipoprotein (ox-LDL) leads to bladder cancer progression through ox-LDL regulation of tumor cell stemness^115^. Using two hypercholesterolemic mouse models (induced by feeding the mice high-fat high-cholesterol chow or knocking out the Ldlr gene), researchers demonstrated that excessive serum cholesterol increased tumor cell stemness and promoted bladder cancer development. In contrast, mice with hormone-induced hypercholesterolemia treated with the selective cholesterol uptake inhibitor ezetimibe showed significantly reduced tumor cell stemness and slower tumorigenesis, suggesting that cholesterol was critical for the increased malignancy of bladder cancer in these mice. Further validation and signaling pathway analyses revealed that ox-LDL binds to the bladder cancer cell membrane receptor CD36 and affects the intracellular JAK2-STAT3 signaling pathway, thereby regulating tumor cell stemness-related genes and promoting bladder cancer cell proliferation.

A comparison of the mRNA expression patterns in patient-derived glioblastoma spherical cells, which maintain stemness, and their differentiated counterparts, which lose stemness, revealed that the majority of the changed genes were networked in cholesterol metabolism pathway^116^. Among these differentially expressed genes, the isoprenoid biosynthesis enzyme farnesyl diphosphate synthase (FDPS) is critical for the survival of glioblastoma stem cells. Although the mechanism is unclear, cholesterol metabolism plays an important role in maintaining cell stemness.

### Cholesterol metabolism and the DNA damage response in cancer cells

Dyshomeostasis of cholesterol metabolism and the DDR are hallmarks of cancer. Few studies have addressed the relationship between cholesterol metabolism and the DNA repair process. However, one study reported that lovastatin, which blocks cholesterol biosynthesis, may inhibit gallbladder cancer (GBC) cell proliferation by attenuating the DNA repair process^117^. This investigation showed that lovastatin sensitized GBC to cisplatin-induced apoptosis by impairing the DDR, which involved inhibiting CHK1, CHK2 and γ-H2AX activation. Treatments using statins with differing chemical properties to eliminate HMGCR or adding MVA reversed cisplatin resistance. RNA sequencing of GBC cells stimulated by statins revealed enrichment with DDR and G2/M checkpoint signaling factors. Treatment with statins or MVA, in fact, inhibited DDR and CHK1 signaling; specifically, DDR activation was suppressed by the inhibition of CHK1 and γ-H2AX expression. Another study revealed that pravastatin, an inhibitor of cholesterol biosynthesis, induced Mdm2 Ser166 phosphorylation, which increased Mdm2 ubiquitin ligase activity, attenuated p53 degradation and inhibited the DDR in hepatocytes^118^. Although a correlation between cholesterol metabolism and the DDR was identified, the mechanism underlying this relationship remains unknown.

## Targeting cholesterol metabolism in cancer therapy

### Targeting cholesterol synthesis for cancer treatment

Many basic studies and several clinical trials have examined the potential therapeutic efficacy of targeting cholesterol metabolism in cancer therapy (Table 2). The potential therapeutic involve targeting cholesterol synthesis, from the inhibition of specific enzymes to the blockade of the feedback mechanism regulated by SREBPs, have been clinically investigated^119^. In some of these studies, statins, which are HMGCR inhibitors, were developed and then used as the standard therapy for patients with high cholesterol levels. Statins induced multiple antitumor effects in tumor cell lines and preclinical animal models through a variety of distinct mechanisms, including the promotion of apoptosis, induction of cell cycle arrest, and inhibition of cell proliferation and invasion^120^. Statins also decreased the cholesterol level and inhibited proteasome action as well as the production of nitric oxide and ROS^121–123^. In addition, statins affected signaling pathways associated with tumorigenesis, including the p53, Akt, mTOR, Myc, p38, and VEGF pathways^42,123–125^. Although early trials reported an increased incidence of cancer in statin drug users, recent studies have shown a 20–28% reduction in the risk of using statins in cancer treatment, and the use of statins has been shown to reduce the risk of recurrence in prostate cancer patients who underwent radical prostatectomy^126,127^. The specific chemical properties of different statins determine their mechanisms of action. Some studies have shown that lipophilic statins are more likely to enter extrahepatic cells, while hydrophilic statins show greater affinity for the liver^128^. Pravastatin is a hydrophilic statin that is characterized as a sodium-independent organic anion translocator expressed only in the liver. In contrast, the lipophilic HMGCR inhibitor simvastatin is a hydrophobic statin that enters cells through various mechanisms. Pravastatin significantly reduces the risk of breast cancer recurrence, whereas hydrophilic statins do not^129,130^. Simvastatin reduces the level of geranylgeranyl pyrophosphate (GGPP) produced in the MVA pathway and the isoprenylation rate of the small GTPase Rab5 in antigen-presenting cells, thereby blocking endosome maturation, prolonging antigen retention, enhancing antigen presentation and T-cell activation, and ultimately enhancing antitumor immunity. In addition, simvastatin potently enhances tumor vaccine efficacy and synergizes with anti-programmed death receptor-1 (PD-1) antibodies in cancer treatment^131^.

**Table 2:** Targeting cholesterol metabolism for cancer therapy.

	Drug	Target	Mechanism	Clinical development	Cancer type	References
Targeting cholesterol synthesis	Statins	HMGCR	Decreases cancer cells mortality and prolongs survival	In Phase I, II and III clinical trials	Various cancers	^42,121–125^
	Simvastatin+EGFR	HMGCR	Blocks endosome maturation, enhancing antigen presentation and T-cell activation	Phase II clinical trials	Non-small cell lung cancer	^135,139^
	Bisphosphonate	FDPS	Increases bone density and reduces skeletal-related complications	Approved for bone metastasis treatment	Various cancers	^140–142^
	RO 40-8071	OSC	Inhibits tumor growth and metastasis	In preclinical development	Colorectal and pancreatic cancer	^143^
	Terbinafine	SQLE	Unknown	In preclinical development		^144^
Targeting cholesterol uptake and cholesterol efflux	U18666A	NPC1	Blocks NPC1-mediated cholesterol export	In preclinical development		^146^
	Itraconazole	NPC1	Blocks NPC1-mediated cholesterol export	In Phase I and II clinical trials	Various cancer	^145^
	Ezetimibe	NPC-1L1	Unknown	In preclinical development		^147^
	Dalcetrapib	CETP		Invalid		^149^
	Evacetrapib	CETP		Invalid		^148^
	Torcetrapib	CETP		Invalid		^150^
	SR9243	LXR	Represses lipogenesis and glycolysis in cancer cells; induces cell apoptosis		CRC, prostate, and lung cancer models	^153,154^
	LXR623	LXR	Decreasing cholesterol levels in cancer cells	In preclinical development	Clear cell renal cell carcinoma model	^154^
	T0901317	LXR	Increasing ABCA1 expression		lung epithelial cell lines	^155^
	RGX-104	LXR	Increasing T-cell activation	In a Phase I clinical trial	B16F10 melanoma and Lewis lung carcinoma models	^156^
Targeting cholesterol esterification	Avasimibe	ACAT-1	Inhibiting proliferation, metastasis, and invasion	In preclinical development	pancreatic and prostate cancer	^157–159^

Statin therapy prolongs the survival of patients with MM, colorectal cancer or metastatic pancreatic cancer who have been treated with a combination of first-line chemotherapeutic agents^132–134^. Perioperative trials with patients in the early stage of invasive breast cancer have shown that high doses of neoadjuvant atorvastatin and fluvastatin reduced tumor proliferation^135,136^. Atorvastatin or fluvastatin also induced MM cell apoptosis by targeting HMGCR^137^. Long-term statin therapy prolonged the survival of patients with glioblastoma multiforme^138^. Notably, the results of Phase II clinical trials for patients with non-small cell lung cancer indicated that a combination of simvastatin and the EGFR inhibitor gefitinib exerted better antitumor effects than treatment with the EGFR inhibitor alone^135,139^. Of course, some conflicting results do not corroborate the findings suggesting statin-conferred protection against cancer. The individual clinical effects of statins may depend on the specific chemical properties of the statin analyzed, the heterogeneity of the tumor treatment, duration of statin use, and other factors.

Other enzymes in the cholesterol biosynthesis pathway have been identified as drug targets. Bisphosphonate, a farnesyl diphosphate synthase (FDPS) inhibitor, has been approved for treating bone metastases because it may improve bone density and thus reduce skeletal-related complications^140–142^. RO48-8071, an inhibitor of oxidative oxytocin cyclase (OSC), significantly inhibits the growth and metastasis of colorectal and pancreatic tumors^143^. Application of this inhibitor increases the cell apoptosis rate and reduces cell proliferation. More importantly, RO48-8071 in combination with 5-fluorouracil showed enhanced antitumor effects. SQLE plays an important role in cholesterol synthesis and has been used as an antitumor target^143^. Several drugs targeting SQLE, such as terbinafine, have been clinically approved as antifungal agents; however, whether they can be used as antitumor agents remains to be investigated^144^.

### Targeting cholesterol uptake and cholesterol efflux in cancer treatment

Numerous small molecules, including the cationic sterol U18666A and a class of triazole antifungal drugs, including itraconazole, can block NPC1-mediated cholesterol export and inhibit DHCR24^145,146^. Ezetimibe is an NPC-1L1 inhibitor that significantly reduces intestinal absorption of cholesterol, decreases plasma cholesterol levels, and promotes cholesterol clearance from plasma^147^. CETP is a plasma glycoprotein synthesized in the liver that binds to HDL and mediates the transfer of cholesterol from HDL to VLDL and LDL, and CETP inhibitors block this process and exert antiatherogenic and cardiovascular risk-reducing effects. Although CETP inhibitors, including torcetrapib, dalcetrapib, and evacetrapib, increase plasma HDL-C levels, these inhibitors are ineffective and cause adverse effects in patients^148–150^. As a result, CEPT inhibitor development has been halted. Niacin, which can increase HDL-C by 20–25% while reducing triacylglycerol and LDL-C levels^151^, has not shown significant tumor-suppressing effects. In diffuse large B-cell lymphoma (DLBCL) cells, mimicking natural HDL in the presence of synthetic HDL nanoparticles, altered cholesterol homeostasis and induced apoptosis^152^. Hence, the effects of HDL-based lipid-lowering drugs on cancer prevention and treatment remain unclear, and further preclinical and clinical trials are needed.

The inverse agonist SR9243 recruits LXR coinhibitors and inhibits LXR activity, thereby inducing cancer cell apoptosis^153^. In addition, the effects of the LXR agonist LXR623 and inverse agonist SR9243 on CCR have been investigated^154^. Both drugs were shown to be effective in inhibiting cancer cell proliferation and inducing apoptosis. SR9243 inhibits lipogenesis mainly by inhibiting some key enzymes, such as acetyl-CoA carboxylase, fatty acid synthase and stearoyl coenzyme A desaturase 1. LXR623 significantly reduces intracellular cholesterol levels by promoting cholesterol efflux and limiting cholesterol uptake. In addition, the LXR agonist T0901317 significantly increased ABCA1 expression in human lung epithelial cell lines^155^. In general, LXR agonists have shown clear results in the treatment of various cancers mainly by inhibiting cancer cell proliferation and inducing apoptosis; however, they can also modulate immune cell activity. RGX-104, an LXR agonist, effectively inhibited the growth of various mouse and human tumors and inhibited myeloid-derived suppressor cell (MDSC) activity by upregulating APOE, which is an LRX target gene, subsequently increasing T-cell activation^156^. Importantly, this LRX activity was further confirmed in a Phase I clinical trial in which an LXR agonist increased T-cell activation.

### Targeting cholesterol esterification in cancer treatment

Avasimibe, an ACAT-1 inhibitor, suppresses cholesterol esterification, increases cellular FC levels and represses tumor metastasis, and the safety of avasimibe has been clinically verified in Phase III clinical trials^157^. In tumor cells, excessive FC can cause ER stress, which can disrupt metabolic homeostasis and lead to apoptosis. Studies have shown that avasimibe-induced inhibition of tumor cell cholesterol esterification effectively inhibited the proliferation, metastasis and invasion of pancreatic and prostate cancer cells^158^. In addition, avasimibe treatment effectively promoted CD8^+^ T-cell function and increased antitumor effects^159^. Cells treated with avasimibe efficiently killed CD19-expressing leukemic cells and promote the secretion of more INF-γ to regulate T-cell cholesterol homeostasis and enhance antitumor effects^160^. Therefore, avasimibe is expected to become an antitumor drug candidate. Studies on the treatment of chronic myelogenous leukemia (CML) have revealed that resistance to imatinib develops after long-term use and that inhibition of BCR-ABL with imatinib or MAPK/cholesterol esterification via avasimibe treatment alone led to insufficient effects. However, combination therapy significantly slowed tumor growth. Specifically, the combination of avasimibe and imatinib synergistically inhibited BCR-ABL mutation- and nonmutation-dependent imatinib CML cell proliferation by targeting cancer-specific CE accumulation, MAPK and natural BCR-ABL signaling^161^. This drug combination, combining relatively nontoxic metabolic inhibitors with existing therapies to overcome cancer cell resistance, is clinically relevant.

## Conclusions

The diagnostic and therapeutic potential of targeting cholesterol metabolism for cancer has recently gained considerable attention. Numerous studies have shown that cholesterol metabolism plays a clear role in tumorigenesis, development, and metastasis; however, many questions remain to be answered. For example, which comes first: cholesterol metabolism disorder or tumorigenesis? Solid evidence for an association between cholesterol and tumorigenesis is lacking. Cholesterol homeostasis is regulated by complex feedback loops, and these mechanisms in tumors remain to be explored. Because of the complexity of the cholesterol metabolic network, inhibition of a single pathway in cholesterol metabolism may exert little therapeutic effect on cancer. Therefore, inhibiting cholesterol metabolism pathways simultaneously or in combination with other signaling pathways is likely to substantially improve cancer treatment. An increasing number of studies have indicated that the crosstalk between factors involved in cholesterol metabolism and TME molecules plays a critical role in tumor growth. Moreover, strategies to develop antitumor inhibitors that regulate the TME and cholesterol level are appealing study topics. Overall, cholesterol metabolism and cancer is an important topic for future anticancer research.
